# Poor effect of osimertinib on *EGFR* exon 20 insertion-positive lung adenocarcinoma: A case report

**DOI:** 10.1097/MD.0000000000022628

**Published:** 2020-10-16

**Authors:** Yuji Inagaki, Akihiro Tamiya, Yoshinobu Matsuda, Kouji Azuma, Yuichi Adachi, Takatoshi Enomoto, Shunichi Kouno, Yoshihiko Taniguchi, Nobuhiko Saijo, Kyoichi Okishio, Shinji Atagi

**Affiliations:** a Department of Internal Medicine; b Department of Psychosomatic Internal Medicine; c Clinical Research Center, National Hospital Organization Kinki-Chuo Chest Medical Center, Sakai, Osaka, Japan.

**Keywords:** EGFR exon 20 insertion, nonsmall cell lung cancer, osimertinib, tyrosine kinase inhibitor

## Abstract

**Introduction:**

The clinical efficacy of osimertinib for patients with lung adenocarcinoma harboring epidermal growth factor receptor (*EGFR*) exon 20 insertion mutations is unclear. Few case reports exist on the successful treatment of such tumors with osimertinib. We report a case wherein osimertinib administration had no effect in a patient with *EGFR* exon 20 insertion-positive lung adenocarcinoma.

**Patient concerns:**

A 48-year-old never-smoking woman was referred to our hospital for chronic cough. Computed tomography (CT) and positron emission tomography-CT revealed a nodule in the right middle lobe, consolidation in the right upper lobe, multiple lymph node metastases, liver metastasis, and multiple bone metastases.

**Diagnosis:**

On the basis of further examination using transbronchial lung biopsy, the patient was diagnosed with cT1N3M1 stage IVB lung adenocarcinoma. An *EGFR* exon 20 insertion, without any additional mutations, was identified.

**Interventions:**

Daily oral administration of 80 mg osimertinib was initiated to treat the *EGFR* exon 20 insertion-positive lung adenocarcinoma.

**Outcomes:**

Although the disease appeared to be stable 2.5 months after the administration of osimertinib, the tumor started to grow 3 months after administration, and carcinoembryonic antigen levels became higher than those before treatment. Thus, osimertinib was discontinued, and treatment with carboplatin as well as pemetrexed and bevacizumab was started, which the patient responded to.

**Conclusion:**

*EGFR* exon 20 insertion mutations must be classified in more detail to assess the efficacy of EGFR tyrosine kinase inhibitors. Osimertinib doses that provide favorable therapeutic windows should be considered. Further clinical research is required to clarify the efficacy of osimertinib and other drugs for exon 20 insertion mutations.

## 1 Introduction

Epidermal growth factor receptor (*EGFR*) mutations are common in never-smoking female patients with lung adenocarcinoma.^[1]^*EGFR* mutations occur most frequently in exons 18 to 21. Patients with exon 19 deletions or exon 21 L858R point mutations show durable responses when treated with EGFR tyrosine kinase inhibitors (TKIs).^[2]^ Unlike the common exon 19 deletions and exon 21 L858R point mutations, exon 20 insertions account for less than 10% of *EGFR* mutations in nonsmall cell lung cancer (NSCLC) patients.^[3]^ These mutations are clustered between codons 762 and 775 and contribute to resistance to gefitinib and erlotinib (first-generation EGFR-TKIs), as well as to afatinib and dacomitinib (second-generation EGFR-TKIs).^[4]^ Recently, osimertinib (a third-generation EGFR-TKI) demonstrated a higher response rate and longer progression-free survival (PFS) than first-generation EGFR-TKIs as first-line chemotherapy.^[5]^ However, the efficacy of osimertinib for patients with lung adenocarcinoma harboring exon 20 insertion mutations is unclear. Osimertinib has been shown to have some activity against adenocarcinomas harboring exon 20 insertion mutations in in vitro and preclinical studies,^[6–9]^ and there are a few case reports on the successful treatment of patients harboring exon 20 insertion mutations with osimertinib. Here, we report a rare case in which osimertinib had a poor effect on *EGFR* exon 20 insertion-positive lung adenocarcinoma.

## 2 Case report

**Figure 1:**
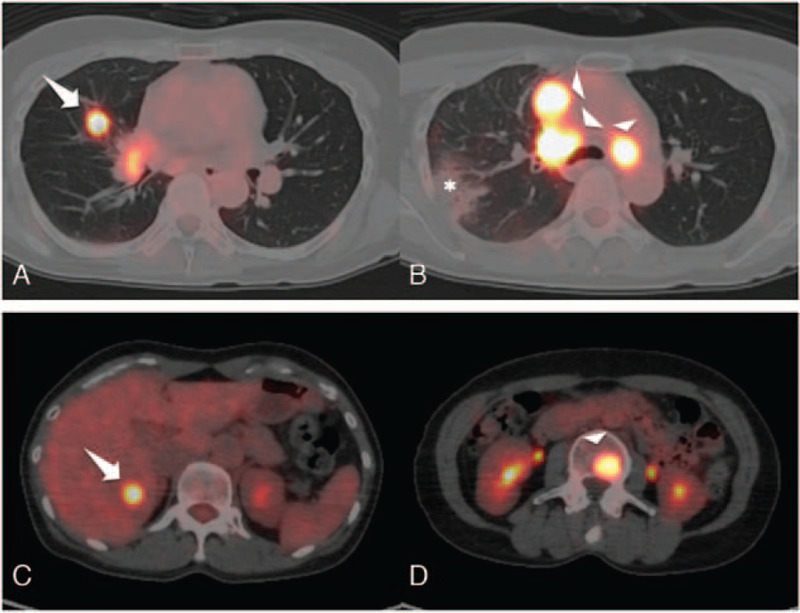
Baseline tumor burden. (A and B) Axial PET-CT image of the thorax revealing a nodule measuring 21 mm in the right middle lobe (larger diameter, arrow), consolidation in the right upper lobe (asterisk), and multiple lymph node metastases (arrowheads). (C and D) PET-CT image of the abdomen showing liver metastasis (arrow) and multiple bone metastases (arrowheads). PET-CT = positron emission tomography-computed tomography.

**Figure 2:**
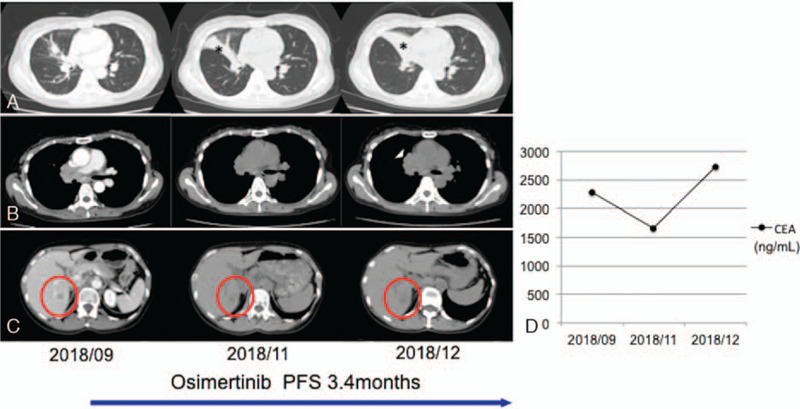
Clinical images and CEA monitoring during the treatment course. (A) CT images of the thorax showing tumor progression and atelectasis after 3.4 months of osimertinib treatment (asterisk). (B) CT images of the thorax showing multiple stable lymph node metastases (arrowhead). (C) CT images of the abdomen revealing a size increase in the liver metastasis (red circle). (D) CEA levels showing disease progression with osimertinib treatment. CEA = carcinoembryonic antigen; CT = computed tomography.

A 48-year-old never-smoking woman was referred to our hospital for chronic cough. Computed tomography (CT) and positron emission tomography-CT revealed a nodule in the right middle lobe, consolidation in the right upper lobe, multiple lymph node metastases, liver metastasis, and multiple bone metastases (Fig. 1). The patient was diagnosed with cT1N3M1 stage IVB lung adenocarcinoma by transbronchial lung biopsy, and her performance status was 1. A real-time polymerase chain reaction *EGFR* mutation assay (COBAS) identified an exon 20 insertion, without additional mutations. We started oral administration of 80 mg/day osimertinib and there was no adverse event. Although a CT scan revealed that the disease was stable 2.5 months after administration of osimertinib, the tumor started to grow 3 months after administration, and carcinoembryonic antigen levels were higher than those before treatment (Fig. 2). We also assessed disease progression 3.4 months after the initiation of osimertinib treatment with Response Evaluation Criteria in Solid Tumors (version 1.1). The treatment was changed to carboplatin (AUC 5) and pemetrexed (500 mg/m^2^) as well as bevacizumab (15 mg/mg); the patient responded to this treatment. The level of alanine aminotransferase, aspartate aminotransferase, and alkaline phosphatase were increased (grade 1) according to National Cancer Institute Common Terminology Criteria of Adverse Events (version 5.0). With a stable disease status, we continued this treatment.

## 3 Discussion

The clinical features associated with an insertion in *EGFR* exon 20 are similar to other *EGFR* mutations in terms of its frequency in female, never-smoking, Asian patients with adenocarcinoma histology.^[1,10–12]^ In the clinical setting, first-generation EGFR-TKIs such as gefitinib and erlotinib are ineffective in patients with exon 20 insertions; second-generation TKIs such as afatinib and dacomitinib induce moderate responses, as opposed to the sensitivity observed in patients with common *EGFR* mutations such as exon 19 deletions and exon 21 L858R point mutations.^[13]^ The response of *EGFR* exon 20 insertions to first-generation EGFR-TKIs has been investigated in retrospective studies. Combining the treatment data of first-generation EGFR-TKIs for the vast majority of exon 20 insertion mutations (other than A763_Y764insFQEA and V769_D770insASV), the overall response rate (ORR) was less than 10%, and the PFS was less than 3 months.^[2,14–18]^ The response of patients with uncommon *EGFR* mutations to afatinib was also investigated in a prospective study.^[13]^ In this study, the ORR was 8.7%, and the PFS was 2.7 months in the exon 20 insertion mutation group. Regarding dacomitinib, only patients with the D770delinsGY mutation in exon 20 showed a partial response.^[19]^ However, because this mutation is extremely rare among exon 20 insertion cases, a positive effect of dacomitinib in patients with exon 20 insertions cannot be extrapolated.^[18]^

Regarding third-generation EGFR-TKIs, osimertinib has shown some activity against exon 20 insertion mutations in preclinical and in vitro studies.^[6,7,9]^ Growth inhibition of tumors harboring the most prevalent exon 20 insertion was observed with osimertinib treatment in xenograft models.^[8]^ However, osimertinib and rociletinib had minimal effect in a patient-derived xenograft model.^[20]^ Two case reports have shown that osimertinib had an effect on exon 20 insertion mutations.^[21,22]^ One patient with stage IV lung adenocarcinoma harboring a rare *EGFR* H773L/V774M mutation complex was administered osimertinib in combination with bevacizumab after a chemotherapy regimen that included a platinum doublet and gefitinib. In this case, osimertinib was effective.^[21]^ In another case, the patient had stage IV lung adenocarcinoma with an insertion [c.2301_2309dup(p.S768_D770dup)] in exon 20. The patient was treated with 160 mg osimertinib daily, and imaging after 8 weeks indicated a partial response. The patient experienced no significant toxicity.^[22]^ Recently, a retrospective study reported that some lung tumors with *EGFR* exon 20 insertion mutations were sensitive to osimertinib.^[23]^ A phase II clinical trial to assess osimertinib as a treatment for *EGFR* exon 20 insertion mutant NSCLC is currently ongoing (NCT03414814). However, the results have not yet been reported. Therefore, the clinical efficacy of osimertinib for patients with lung adenocarcinoma harboring exon 20 insertion mutations remains unclear.

Here, we report a rare case in which a patient with a lung adenocarcinoma harboring an insertion in *EGFR* exon 20 did not respond to osimertinib. We selected osimertinib because preclinical studies have reported that it was effective against lung cancer cells with exon 20 insertions.^[6–9]^ There are 2 possible reasons for this lack of response. First, the lack of response may be because of the heterogeneity of exon 20 insertion mutations. *EGFR* mutations are mostly clustered in the loop region (Ala767-Val774) after the C-helix of the EGFR kinase domain, which is required to regulate the ATP binding cleft; EGFR-TKIs bind to this same location.^[21]^ However, exon 20 insertions can occur in multiple places, and the location of the insertion may affect both the drug mechanism of action and the ATP binding capacity, which ultimately determine resistance or sensitivity to EGFR inhibitors.^[18]^ For instance, the A763_Y764insFQEA mutation is an important exon 20 insertion that has a high in vitro affinity for gefitinib and is sensitive to erlotinib in an engineered cell line model.^[24]^ Moreover, preclinical reports have shown that the location of the insertion may determine the sensitivity to osimertinib.^[9,25]^ More specifically, exon 20 insertion mutations sensitive to osimertinib might be identified. Unfortunately, details on the specific exon 20 insertion mutation of this patient are unknown, owing to limitations in the *EGFR* mutation assay used. Second, the dose of osimertinib used (80 mg daily) might have been too low to treat this pathology considering that, as compared with exon 19 deletions and exon 21 L858R point mutations, most *EGFR* exon 20 insertions need higher concentrations of erlotinib, gefitinib, and afatinib to achieve growth suppression in in vitro models.^[24]^ Considering the toxicities associated with these drugs, it would be difficult to achieve the concentration necessary to inhibit cancer cell growth in tumors harboring exon 20 insertion mutations.^[22]^ However, unlike other EGFR-TKIs, therapeutic plasma concentrations of osimertinib can be achieved, particularly when it is administered at high doses.^[13]^ A phase II study on the daily administration of 160 mg osimertinib in patients with exon 20 insertion mutations is currently ongoing (NCT03191149). In addition, other clinical trials for exon 20 insertion mutations are in progress.^[2]^ Poziotinib has achieved a 64% ORR in a phase II clinical trial (NCT03066206),^[6]^ and the efficacy of other EGFR-TKIs or *EGFR* exon 20 insertion-selective inhibitors is being evaluated.^[18]^ We expect that these drugs will show good efficacy in patients with exon 20 insertion mutations. It is important to find therapeutic agents that consider the molecular heterogeneity of *EGFR* exon 20 mutations.

To the best of our knowledge, there are few reports on osimertinib treatment for NSCLC patients harboring exon 20 insertion mutations. The efficacy of osimertinib was limited in this case. The heterogeneity of exon 20 insertion mutations and the optimal osimertinib dose should be considered to obtain a favorable therapeutic window. Therefore, further clinical research is required to clarify the efficacy of osimertinib and other drugs in lung tumors harboring exon 20 insertion mutations.

## Acknowledgment

We would like to thank the patient, her family, and all of her caregivers.

## Author contributions

**Conceptualization:** Yuji Inagaki, Akihiro Tamiya, Yoshinobu Matsuda, Kouji Azuma, Yuichi Adachi, Takatoshi Enomoto, Shunichi Kouno, Yoshihiko Taniguchi, Nobuhiko Saijo, Kyoichi Okishio, Shinji Atagi.

**Supervision:** Shinji Atagi.

**Writing – original draft:** Yuji Inagaki.

**Writing – review & editing:** Yuji Inagaki, Akihiro Tamiya, Yoshinobu Matsuda, Kouji Azuma, Yuichi Adachi, Takatoshi Enomoto, Shunichi Kouno, Yoshihiko Taniguchi, Nobuhiko Saijo, Kyoichi Okishio, Shinji Atagi.
